# Extracellular Vesicles and Their Renin–Angiotensin Cargo as a Link between Metabolic Syndrome and Parkinson’s Disease

**DOI:** 10.3390/antiox12122045

**Published:** 2023-11-26

**Authors:** Maria A. Pedrosa, Carmen M. Labandeira, Nerea Lago-Baameiro, Rita Valenzuela, Maria Pardo, Jose Luis Labandeira-Garcia, Ana I. Rodriguez-Perez

**Affiliations:** 1 Cellular and Molecular Neurobiology of Parkinson’s Disease, Research Center for Molecular Medicine and Chronic Diseases (CIMUS), Instituto de Investigación Sanitaria de Santiago de Compostela (IDIS), University of Santiago de Compostela, 15782 Santiago de Compostela, Spain; mary.pedrosa@usc.es (M.A.P.); rita.valenzuela@usc.es (R.V.); 2 Networking Research Center on Neurodegenerative Diseases (CIBERNED), 28029 Madrid, Spain; 3 Neurology Service, University Hospital of Ourense, 32005 Ourense, Spain; carmen.maria.labandeira.guerra@sergas.es; 4 Grupo Obesidómica, Área de Endocrinología, Instituto de Investigación Sanitaria de Santiago de Compostela (IDIS), Complexo Hospitalario Universitario de Santiago de Compostela/SERGAS, 15706 Santiago de Compostela, Spain; nerealagobaameiro@gmail.com (N.L.-B.); maria.pardo.perez@sergas.es (M.P.); 5 CIBER Fisiopatología Obesidad y Nutrición, Instituto de Salud Carlos III, 28029 Madrid, Spain

**Keywords:** adipocytes, angiotensin receptor blockers, exosomes, NADPH-oxidase, neurodegeneration, neuroinflammation, obesity, oxidative stress

## Abstract

Several studies showed an association between metabolic syndrome (MetS) and Parkinson’s disease (PD). The linking mechanisms remain unclear. MetS promotes low-grade peripheral oxidative stress and inflammation and dysregulation of the adipose renin–angiotensin system (RAS). Interestingly, brain RAS dysregulation is involved in the progression of dopaminergic degeneration and PD. Circulating extracellular vesicles (EVs) from MetS fat tissue can cross the brain–blood barrier and may act as linking signals. We isolated and characterized EVs from MetS and control rats and analyzed their mRNA and protein cargo using RT-PCR and the ExoView R200 platform, respectively. Furthermore, cultures of the N27 dopaminergic cell line and the C6 astrocytic cell line were treated with EVs from MetS rats. EVs were highly increased in MetS rat serum, which was inhibited by treatment of the rats with the angiotensin type-1-receptor blocker candesartan. Furthermore, EVs from MetS rats showed increased pro-oxidative/pro-inflammatory and decreased anti-oxidative/anti-inflammatory RAS components, which were inhibited in candesartan-treated MetS rats. In cultures, EVs from MetS rats increased N27 cell death and modulated C6 cell function, upregulating markers of neuroinflammation and oxidative stress, which were inhibited by the pre-treatment of cultures with candesartan. The results from rat models suggest EVs and their RAS cargo as a mechanism linking Mets and PD.

## 1. Introduction

The exact pathogenic mechanisms of Parkinson’s disease (PD) are not fully understood. However, it is currently accepted that neuroinflammation and oxidative stress are major factors in dopaminergic neuron degeneration [1,2]. Several studies have shown an association between systemic inflammatory processes and neuroinflammation, including, those showing a low level of peripheral oxidative stress and inflammation such as aging (i.e., “inflammaging” [3], menopause [4,5], and metabolic syndrome [6,7,8]. However, the possible mechanisms linking peripheral processes with neuroinflammation, and neurodegeneration remain to be clarified.

Metabolic syndrome (MetS) is a complex disorder characterized by at least three of the following criteria: abdominal obesity, insulin resistance, hypertension, hypercholesterolemia, and hypertriglyceridemia [9]. MetS promotes peripheral oxidative stress [10], low-grade chronic peripheral inflammation [11], and dysregulation of the adipose and circulating Renin–Angiotensin System (RAS) towards its pro-oxidative pro-inflammatory axis (see below) [12]. In both human and animal models, the dysregulation of the circulating RAS is positively correlated with body fat tissue [13], and brain RAS dysregulation is involved in the progression of dopaminergic degeneration and PD [14,15,16].

The RAS was initially considered a circulating/hormonal system related to the control of blood pressure; then, the presence of a local/paracrine RAS was observed in most tissues, including brain tissue and fat tissue [17,18], and tissue RAS was observed to play a major role in regulating tissue oxidative stress and inflammation [17,18]. Angiotensin II (Ang II) is the most important RAS effector peptide, which is formed from the precursor protein angiotensinogen (AGT) by the sequential actions of two enzymes, renin/prorenin (PR) and angiotensin-converting enzyme (ACE), and Ang II acts on two major receptors: Ang II type 1 (AT1) and type II (AT2) receptors. Several additional RAS components contribute to RAS regulation, such as ACE2 and its product Ang (1–7), which acts on MAS receptors, and PR acting on PR receptors (PRR). Altogether, RAS is organized into two major axes: a pro-oxidative/pro-inflammatory arm, mainly formed by the Ang II/AT1 and PR/PRR, and an anti-oxidative/anti-inflammatory arm mainly formed by Ang II/AT2 and ACE2/Ang (1–7)/MAS [17,18,19]. Interestingly, astrocytes are the most abundant cell in the brain and are both the main source of brain AGT [20,21,22] and major players in PD progression [15,23].

Circulating extracellular vesicles (EVs) may act as inflammatory mediators [24,25] and as oxidative stress signal carriers [26]. We will use the term EVs to refer to conventionally called exosomes with a diameter of 40–150 nm [27]. Due to their genesis, EV cargo reflects the physiological status of the origin cell [28]. MetS induces increased production and release of adipocyte-derived EVs, which are characterized by the expression of perilipin A [29,30] and caveolin [31]. A major role of RAS components in EV cargo was observed in the progression of chronic renal disease associated with diabetes [32], in vessel alterations associated with hypertension [33], and in heart pathology induced by Ang II [34]. Interestingly, EVs can cross the blood–brain barrier (BBB) in a bi-directional manner [35,36]. However, the possible effects of MetS EV RAS cargo on dopaminergic neuron degeneration and astrocytic function are not known. In the work reported here, we first studied whether EVs from MetS rats show dysregulation of RAS cargo. Then, we studied the possible effects of MetS EVs RAS components on a cultured dopaminergic N27 cell line, particularly the effects of RAS dysregulation on dopaminergic N27 cell line vulnerability, and the effects of MetS EVs on cultured C6 astrocytic cell line as the main cell source of brain angiotensin. The possible effects of MetS EVs on interactions between neurons, astrocytes, and microglia will be addressed in future studies designed from the present results.

## 2. Materials and Methods

### 2.1. Animal Experiments

Young adult male Sprague Dawley rats (2–3-month-old; males) without (controls) and with metabolic syndrome (MetS; obesity, increased blood pressure, hyperglycemia) were used in the present study. Male rats were used because it is known that female rodents are protected against high-fat diet (HFD)-induced metabolic changes [37]. Rats used for MetS (*n* = 6) received a HFD with 60% of calories from fat (D18042603; Research Diets, USA) for a period of 18 weeks as was previously described [7]. A group of rats with MetS (*n* = 6) was treated with the AT1 receptor blocker Candesartan (AstraZeneca, Cambridge, UK). Powered candesartan was administered orally mixed with “Nocilla” hazelnut cream (Nutrexpa, Barcelona, Spain). Animals not treated with candesartan were given “Nocilla” hazelnut cream only. The dose of candesartan (candesartan cilexetil, 1 mg/Kg/day for at least 4 weeks) was based on the results of our previous studies [16]. A group of animals receiving a normal diet was used as control group (*n =* 6).

All animal experiments were carried out in accordance with Directive 2010/63/EU, European Council Directive 86/609/EEC, and Spanish legislation (RD53/2013). Animal experiments were approved by the corresponding committee at the University of Santiago de Compostela (15005/15/002) and were carried out in the Experimental Biomedicine Centre (CEBEGA; University of Santiago de Compostela). Rats were housed at a constant room temperature (RT) (21–22 °C) and 12 h light/dark cycle. Blood extraction was performed by decantation at the moment of sacrifice. Blood was kept at RT between collection and centrifugation for no more than 30 min to minimize the release of platelet EVs that occurs under cold temperatures, agitation, and prolonged storage of blood [38]. Then, the serum was separated by centrifugation at 2000× *g* for 10 min. Finally, aliquots of serum samples were stored at −80 °C until processed.

EVs were isolated, characterized, and their mRNA and protein cargo determined as detailed below. mRNA cargo for RAS components, and pro-oxidative and pro-inflammatory markers were determined using RT-PCR analysis. Protein cargo for RAS components was determined using the interferometric imaging platform ExoView^®^ R200 (NanoView Biosciences, Boston, MA, USA), where total serum EVs were captured using tetraspanin chips, and EVs enriched from fat tissue origin were captured using the caveolin/perilipin method. In a second series of experiments, cultures of the N27 dopaminergic cell line and the C6 astrocytic cell line were treated with EVs from MetS rats to know if EVs can be uptaken by N27 dopaminergic cells and the C6 astrocytic cell line, and if EVs affect dopaminergic N27 cell line degeneration, and markers of neuroinflammation, oxidative stress, and RAS activity in the recipient cells. Parallel and preliminary experiments revealing no significant effects of EVs from control rats (EV_Control_) are shown in the corresponding figures and Figure S1.

### 2.2. Isolation of EVs from Animal Serum

EVs were isolated from 500 µL of serum using the ExoQuick^®^ (EXOQ20A-1, System Biosciences, Palo Alto, CA, USA) precipitation method according to the manufacturer’s instructions. Briefly, serum samples were centrifuged at 3000× *g* for 10 min at 4 °C to remove cell debris. Then, 126 µL of ExoQuick^®^ precipitation buffer was added and incubated for 30 min at 4 °C without shaking. Next, the sample was centrifuged at 1500× *g* for 30 min and the supernatant was discarded. The obtained pellet (enriched in EVs) was resuspended in phosphate-buffered saline (PBS).

### 2.3. EV Characterization by Western Blot, Transmission Electron Microscopy (TEM), and Esterase Activity

A first characterization of the EVs was carried out using Western blot, transmission electron microscopy (TEM), and by quantifying enzymatic esterase activity within the vesicles [39,40]. For the analysis of EV markers, an equal amount of EV protein lysates was separated on 8–12% Bis-Tris polyacrylamide gel and transferred to nitrocellulose membranes. Membranes were incubated overnight at 4 °C with primary antibodies to CD63 (MX-49.129.5; sc-5275, Santa Cruz Biotechnology, Santa Cruz, CA, USA), to CD9 (EPR2949; ab92726, Abcam, Cambridge, UK), and to CD81 (EPR4244, ab109201, Abcam). The antibody against calnexin (10427-2-AP, Proteintech, Manchester, UK) was used as a negative control. The following horseradish peroxidase (HRP)-conjugated secondary antibodies were used: goat anti-rabbit-HRP (sc-2004; Santa Cruz Biotechnology; 1:2500) and goat anti-mouse-HRP (sc-2031, Santa Cruz Biotechnology; 1:2500). Bound antibodies were detected with an Immun-Star HRP Chemiluminescent Kit (Bio-Rad; 170–5044, Hercules, CA, USA) and visualized with a chemiluminescence detection system (Bio-Rad; Molecular Imager ChemiDoc XRS System). For transmission electron microscopy (TEM), enriched EVs were diluted 1:10 in distilled water. Then, 10 μL was loaded on a carbon-coated copper grid for 1 min and the excess sample suspension was blotted with filter paper. The grid was placed on a drop of 1% phosphotungstic acid solution in Milli-Q^®^ H_2_O for 1 min, and the excess stain was removed. Finally, the morphology of the EVs was examined using a JEOL JEM 2010 transmission electron microscope at 200 Kv. For esterase activity, an equal amount of enrichment of EV protein lysates was used for the EXOCET exosome quantification assay (EXOCET96A-1, System Biosciences) according to the manufacturer’s instructions.

### 2.4. EV-RAS Protein Analysis by Exoview^®^

To analyze EVs without contaminants, we used the new single particle interferometric reflectance imaging sensor (SP-IRIS) ExoView^®^ R200 (now Leprechaun from Unchained labs, Pleasanton, CA, USA) which allows us to carry out EV particle size analysis and measure the EV concentration, EV phenotype, and biomarker colocalization. This technology is based on the single particle interferometric reflectance imaging sensor technique, which has previously been used to detect viruses and EVs down to 50 nm in size [41]. Complete characterization of serum-derived EVs (*n* = 6/per group) was performed using ExoFlex Tetraspanin chips (NAVEV-TM-FLEX, Nanoview Biosciences). Chips were conjugated following the ExoFlex conjugation protocol (Nanoview Biosciences). To analyze the EVs from fat tissue, Perilipin A (NB110-40760, Novus Biologicals, Centennial, CO, USA) and Caveolin-1 (NB10-615, Novus Biologicals) were used as antibody linkers. We selected these markers based on previous studies that identified Perilipin A (perilipin-1) as a specific marker to discern EVs from adipocyte origin from others at a circulating level [30], which was later confirmed by other researchers [42]. Regarding caveolin, proteomic analysis studying the cargo of EVs released by adipocytes showed a high content of caveolin-1, which can be used as adipocyte EV marker [31,43].

Chips were pre-scanned following the instructions provided by the manufacturer’s protocol to generate baseline measurements of pre-adhered particles before sample incubation. For sample incubation, 50 µL of EVs (diluted at 1:25 in incubation solution buffer) were carefully loaded on the pre-scanned chip and incubated overnight at room temperature without agitation. The incubation was carried out in sealed 12-well plates. Then, several washes were performed according to the manufacturer’s protocol.

The fluorescent labeling of detection antibodies was performed with Alexa Fluor Conjugation Kits; specifically, we used the commercial ACE2 antibody (MAB9332, Bio-Techne R&D Systems, Minneapolis, MN, USA) labeled with 488-fluor (ab236553, Abcam), MasR antibody (AAR-013, Alomone labs, Jerusalem, Israel) labeled with 647-fluor (ab269823, Abcam), and PRR antibody (ab40790; Abcam) labeled with 594-fluor (ab269822, Abcam). The specificity of these antibodies was previously assessed in our laboratory by immunoblot analysis of lysates from HEK293 cells transiently transfected with the corresponding GPCR tagged to fusion tail DDK (i.e., a C-terminal DDK epitope tag DYKDDDDK) or GFP (green fluorescent protein) [44,45], or was KO validated by the supplier. Due to the absence of specific commercial antibodies for the AT1 receptor, we used autoantibodies against the AT1 receptor purified by affinity chromatography from preeclamptic women’s serum samples, as we previously described [46], which were labeled with 755-fluor (ab201805, Abcam). Women’s serum samples were obtained in the Obstetric Service of the University Hospital Complex of Santiago de Compostela. All patients were informed of the study’s purpose and protocol and signed an informed consent. Approval for this study was obtained from the Galician Drug Research Ethics Committee (CEIm-G), protocol 2017/618, and the research was carried out in accordance with the principles of the Helsinki Declaration. Due to the lack of confirmed specific commercial antibodies against angiotensinogen that can be used for the ExoView, the characterization of this RAS component was only based on EV mRNA cargo (see below). Finally, fluorescently labeled antibodies for ACE2 (1.5 µg/µL), MasR, PRR, and AT1 (0.6 µg/µL) were incubated for 1 h with gentle agitation for detection of these RAS components in EV cargo. Then, following the manufacturer´s instructions, the chips were washed with different solutions and Milli-Q water for final analysis using the ExoView scanner 3.2.1 acquisition software. Images acquired were analyzed using ExoView Analyzer 3.1.4 software (Nanoview Biosciences; now Unchained labs). Number and size data were obtained by means of the total particle number captured by tetraspanins and by fat tissue antibody linkers. Fluorescence intensity data were normalized by the number of particles captured by each of the antibody linkers.

### 2.5. EV-mRNA Cargo Analysis

Total EV-RNA from 500 µL of serum was isolated using an exoRNeasy Midi Kit (77164, Qiagen, Hilden, Germany) according to the manufacturer’s protocol described in the exoRNeasy Serum/Plasma Handbook. Total RNA was reverse transcribed to complementary DNA using the high-capacity RNA-to-cDNA kit (4387406, Applied Biosystems). The RT-PCR analysis was performed using a QuantStudio 3 platform (Applied Biosystems, Foster City, CA, USA). The EvaGreen qPCR MasterMix (Applied Biological Materials Inc., Vancouver, BC, Canada), and the corresponding primer sequences (see below) were used to examine the relative levels of ACE2, AT1, PRR, AGT, and Mas receptors. GAPDH was used as a housekeeping gene and was amplified in parallel with the genes of interest. We used the comparative cycle threshold values (cycle threshold (Ct)) method (2^−ΔΔCt^) to examine the relative messenger RNA (mRNA) cargo. A normalized value was obtained by subtracting the Ct of GAPDH from the Ct of interest (ΔCt). As it is uncommon to use ΔCt as a relative expression data due to this logarithmic characteristic, the 2^−ΔΔCt^ parameter was used to express the relative expression data. Table 1 summarizes the primer sequences for the target genes.

### 2.6. Cultures of the N27 Dopaminergic Cell Line and the C6 Astrocytic Cell Line

Cultures of the N27 dopaminergic cell line and the C6 astrocytic cell line were carried out as previously described [47]. The N27 dopaminergic cell line derived from rat mesencephalic tissue (SCC048, Millipore, MA, USA) was cultured in RPMI 1640 medium supplemented with 10% FBS, 2 mM L-glutamine (Sigma-Aldrich, St. Louis, MO, USA), 100 U/mL penicillin, and 100 μg/mL streptomycin. The C6 astroglial cells (CB_92090409, Sigma-Aldrich) were cultured in Ham’s F12 medium with 10% FBS, 2 mM L-Glutamine (Sigma-Aldrich), 100 U/mL penicillin, and 100 μg/mL streptomycin. All cultures were maintained at 37 °C and 5% CO_2_ in a humidified incubator in a 75 cm^2^ culture flask. Once cells became confluent, they were re-seeded onto 12-well plates (Falcon, Becton Dickinson, Franklin Lakes, NJ, USA) at a density of 0.5 × 10^5^ cells/cm^2^. We confirmed the expression of the dopaminergic marker TH (tyrosine hydroxylase) and the astrocytic marker GFAP (glial fibrillary acidic protein) by cultured N27 and C6 cells [20], respectively (Figure S1).

### 2.7. EV Labeling and Uptaking by the N27 Dopaminergic Cell Line and the C6 Astrocytic Cell Line

Serum EVs from animals suffering MetS (EV_MetS_) were labeled with the ExoGlow™-Protein EV labeling kit (EXOGP100A-1, System Biosciences) following the manufacturer´s instructions. Cultures, (the N27 dopaminergic cell line and the C6 astrocytic cell line) were treated with labeled EV_MetS_ for 12 h. Then, cell line cultures were fixed with 4% paraformaldehyde in Dulbelcco’s phosphate-buffered saline (DPBS, pH 7.4) for 20 min and, after three washes, incubated overnight at 4 °C with a mouse monoclonal Anti-β-Actin antibody (1:1500; A2228, Sigma-Aldrich) as a cytoplasmatic marker. Cultures were then rinsed with DPBS and incubated for 2 h at room temperature with the secondary antibody Alexa Fluor 568-conjugated donkey anti-mouse IgG (1:200; A10037, Invitrogen), and then incubated for 15 min with the DNA-binding dye Hoechst 33342 (3 × 10^−5^ M in DPBS; Sigma) to visualize cell nuclei. Finally, cultures were coverslipped with Immu-Mount (9990402, Thermo Scientific™ Shandon™, Waltham, MA, USA) and the immunolabeling was visualized using confocal microscopy (model TCS SP8; Leica, Wetzlar, Germany).

### 2.8. Culture Treatments and MTT Assay

The N27 dopaminergic cell line and C6 astroglial cell line cultures were treated with an EV suspension vehicle: phosphate-buffered saline (PBS; i.e., control group) or with EV_Control_ (i.e., EVs from control rats) or EV_MetS_ (i.e., EVs from MetS rats, 1 µg/µL) alone or EV_MetS_ (1 µg/µL) plus the angiotensin type I receptor blocker candesartan (1 μM; 4791, Tocris, Bristol, UK) for 24 h. To study the mRNA expression of RAS components and receptors and inflammatory and oxidative markers, cells were collected and analyzed by RT-qPCR. The N27 dopaminergic cell line cultures were used to study the effects of serum EVs from MetS on neurodegeneration triggered by the dopaminergic neurotoxin 6-hydroxydopamine (6-OHDA). The cultures were exposed to 6-OHDA alone (40 μM; based on the results of previous studies using 6-OHDA in N27 dopaminergic cell line cultures [48]) or EV_MetS_ (1 µg/μL) alone or EV_Contro_l (1 µg/μL) alone or 6-OHDA plus EV_MetS_ (1 µg/μL) or 6-OHDA plus EV_MetS_ plus the angiotensin type I receptor blocker candesartan (1 μM; 4791, Tocris) or candesartan alone for 24 h. To evaluate cell viability, the 3-(4,5-dimethylthiazol-2-yl)-2, 5-diphenyltetrazolium bromide (MTT) assay was performed [46]. This colorimetric assay is based on the conversion of yellow MTT to purple formazan crystals in living cells by mitochondrial dehydrogenases. Briefly, 20 μL of MTT (5 mg/mL; Sigma-Aldrich) were added to each well. After incubation at 37 °C for 4 h, the medium was removed, and the MTT-formazan product was extracted with 50 μL of acidic isopropanol (0.04 mol/L). After 30 min at room temperature, the absorbance of the formazan solution was measured with an Infinite M200 multiwell plate reader (TECAN) at 570 nm with a reference wavelength of 690 nm.

### 2.9. NADPH-Oxidase Activity and Cytokine Levels

The NADPH-oxidase (NOX) activity of C6 astroglial cells and the N27 dopaminergic cell line was measured by lucigenin-enhanced chemiluminescence in an Infinite M200 multiwell plate reader (TECAN). NOX activity was measured on the cell lysates in the presence of NADPH-oxidase substrate (10^−4^ mol/L; Sigma) and lucigenin (5 × 10^−6^ mol/L; Sigma). The reaction was performed using 20–50 μg of protein as described by [49,50]. Chemiluminescence was expressed as relative light units (RLU/mg protein × min).

Cytokine levels were measured in cell culture supernatants (*n* = 6 per group). Culture mediums were collected and centrifuged at 3000× *g* for 10 min to eliminate cell debris. Then, the supernatants were used to measure cytokine levels using specific ELISA, according to the manufacturer’s instructions: for rat TNF-α (Diaclone 865.000.96, Gen-Probe Diaclone SAS); for rat IL-6 (MBS701221, MyBioSource, San Diego, CA, USA); and for rat IL-1 β (MBS284210, MyBioSource).

### 2.10. Analysis of Cell mRNA Expression in Recipient Cells

Total RNA from cell homogenates was extracted with TRIzol (Invitrogen, Paisley, UK) following the manufacturer’s protocol. Total RNA (2 μg) was reverse transcribed to complementary DNA (cDNA) using nucleoside triphosphate containing deoxyribose, random primers and Moloney murine leukemia virus (MMLV; Invitrogen, Thermo Fisher Scientific, Waltham, MA, USA; 200U) reverse transcriptase. The RT-PCR analysis was performed as described in the EV-mRNA cargo analysis. The primer sequences are summarized in Table 1.

### 2.11. Statistical Analysis

Statistical analyses were performed using SigmaPlot 11.0 (Systat Software, Inc., San Jose, CA, USA). Data normality was tested with the Kolmogorov–Smirnov test. Parametric tests were used when the dataset passed the normality test: Student’s *t*-test for two group comparisons and one-way ANOVA followed by the Student–Newman–Keuls method for multiple comparisons. For nonparametric data, multiple comparisons were carried out by Kruskal–Wallis one-way analysis of variance on ranks test followed by Student–Newman–Keuls or Dunn’s methods as post hoc tests. All data were expressed as mean ± SEM. Differences were considered statistically significant at *p* < 0.05. GraphPad Prism 8 software (GraphPad Inc., San Diego, CA, USA) was used to create scatter dot plot graphs. All statistics are shown in Table S1 in the Supplementary Materials.

## 3. Results

### 3.1. Isolation and Characterization of EVs from Rat Serum and Effects of MetS and Candesartan Treatment

**Figure 1:**
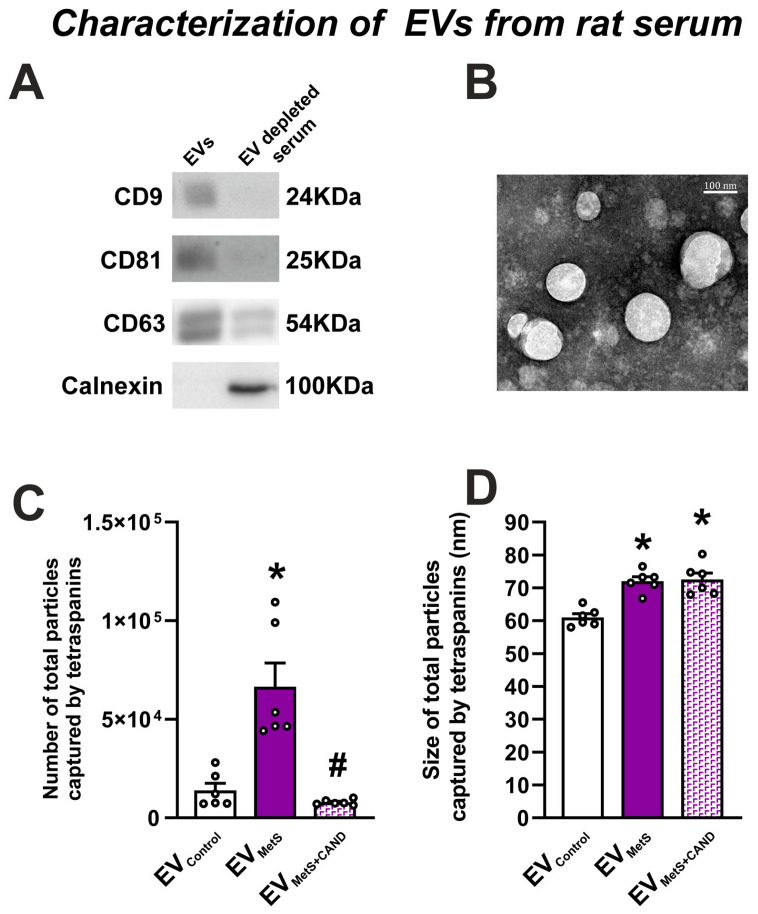
Expression of specific EV markers (CD9, CD81, CD63) and negative EV markers (calnexin) was confirmed by Western blotting in ExoQuick isolated samples (**A**). Transmission electron microscopy (TEM) visualization of EV_MetS_ isolated using ExoQuick (**B**). Using the Exoview platform chip, captured particles from animal serum were increased in MetS rats and significantly decreased when MetS rats were treated with candesartan (**C**). Captured particles from MetS rats showed a larger size than those captured from control animals (**D**). Scale bar: 100 nm. Data are given as the mean ± SEM. * *p* < 0.05 relative to the control group; # *p* < 0.05 relative to the EV_MetS_. (Kruskal–Wallis one-way ANOVA with the Student-Newman-Keuls method as a post hoc test (**C**); one-way ANOVA with the Student-Newman-Keuls method as a post hoc test (**D**)). CAND: candesartan; EVs: extracellular vesicles; EV_Control_: EV_S_ isolated from control animals; EV_MetS_: EVs isolated from the serum of metabolic syndrome animals; EV_MetS+CAND_: EVs isolated from the serum of metabolic syndrome animals treated with candesartan; MetS: metabolic syndrome.

EVs were isolated from 500 µL of serum, obtained from control and MetS rats using ExoQuick. The expression of specific EV markers (CD9, CD81, CD63) was confirmed by Western blotting. The expression of all markers was increased in the EV-enriched sample compared to the EV-depleted serum sample from the same animal (Figure 1A). In contrast, the expression of the calnexin marker, used as a negative control, was not observed in the EV-enriched sample (Figure 1A). Furthermore, TEM showed the presence of purified EVs with round structures with a 70 nm diameter (Figure 1B) in the EV-enriched sample. We also characterized EV samples using a colorimetric assay that measures the activity of Acetyl-CoA Acetylcholinesterase, an enzyme that is highly expressed in EVs [51,52]. The assay revealed high enzymatic activity in the EV samples, which was significantly higher (*p* = 0.017) in samples from MetS rat serum (0.695 ± 0. 0573 absorbance units (AU); mean ± SEM) than in control rat serum (0.521 ± 0. 0200 AU).

The increase in the number of EVs from MetS rat serum (EV_MetS_) was confirmed by using the single particle interferometric reflectance imaging sensor (SP-IRIS) platform ExoView R200, which corroborated that the number of captured EVs was significantly higher in serum from MetS rats (6.65 × 10^4^ ± 1.21 × 10^4^) relative to control animals (1.39 × 10^4^ ± 3.58 × 10^3^) (Figure 1C). Interestingly, MetS rats treated with the AT1 receptor blocker candesartan for four weeks showed a significant reduction in the number of EVs captured by the ExoView (7.93 × 10^3^ ± 563.44) (Figure 1C). Furthermore, EV_MetS_ were larger (72.04 ± 1.32 nm) than those obtained from serum samples from control rats (EV_Control_) (61 ± 1.12 nm). However, treatment with candesartan did not change the size of EV_MetS_ (72.58 ± 1.94 nm) (Figure 1D).

### 3.2. mRNA Expression of RAS Components in Serum EVs and Effects of Treatment of MetS Rats with Candesartan

**Figure 2:**
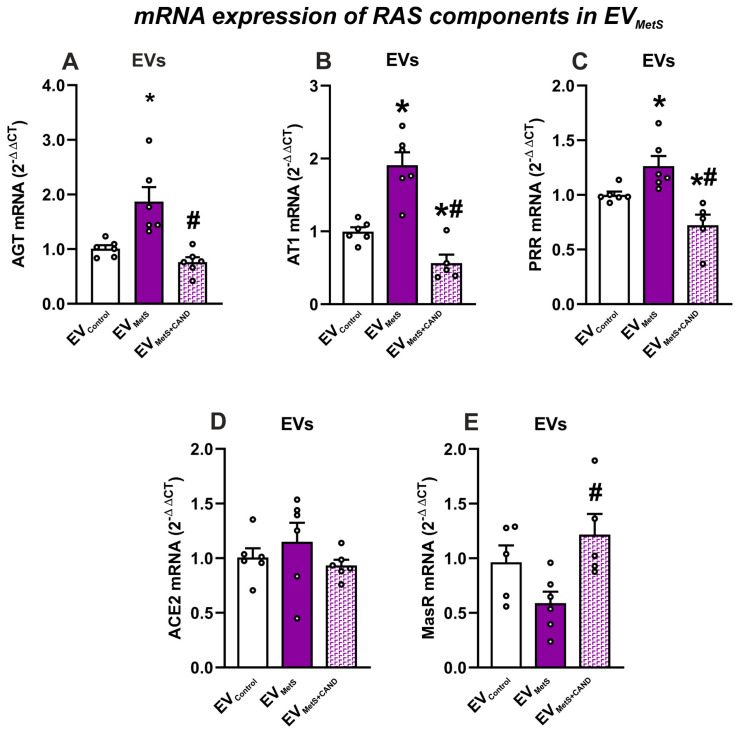
mRNA-cargo of RAS components in EVs. Expression of pro-inflammatory components: AGT (**A**), AT1 (**B**), and PRR (**C**) was increased in EV_MetS_ relative to EV_Control_, which was inhibited when MetS animals were treated with the AT1 receptor blocker candesartan. The expression of the anti-inflammatory component MasR (**E**) was increased in EV_MetS+CAND_. For mRNA, the comparative cycle threshold values method (2−^ΔΔCt^) was used. Data are given as the mean ± SEM. * *p* < 0.05 compared to the EV_Control_ group; # *p* < 0.05 compared to the EV_MetS_ group (Kruskal–Wallis one-way ANOVA with the Student-Newman-Keuls Method as a post hoc test (**A**); one-way ANOVA with the Student-Newman-Keuls Method as a post hoc test (**B**–**E**)). ACE2: angiotensin-converting enzyme 2; AGT: angiotensinogen; AT1: angiotensin type 1 receptor, CAND: candesartan; EVs: extracellular vesicles; EV_Control_: EVs isolated from the serum of control animals; EV_MetS_: EVs isolated from the serum of metabolic syndrome animals; EV_MetS+CAND_: EVs isolated from the serum of metabolic syndrome animals treated with candesartan; MasR: Mas-related receptor; MetS: metabolic syndrome; PRR: (pro)-renin receptor.

The mRNA-cargo of RAS components was characterized in total serum EVs. The mRNA of the pro-inflammatory components AGT and AT1 and PR receptors was significantly higher in EV_MetS_ relative to EV_Control_ (Figure 2A–C). Interestingly, treatment of MetS rats with candesartan promoted a significant decrease in the mRNA cargo of these pro-inflammatory components (AT1 and PRR mRNA expression) in EVs from MetS animals treated with candesartan (EV_MetS+CAND_) relative to EV_MetS_ and even to EV_Control_ (Figure 2A–C). No significant difference was observed in mRNA level of ACE2 and MasR in EV_MetS_ compared to EV_Control_. However, candesartan treatment of MetS rats induced an increase in MasR mRNA levels in EV_MetS+CAND_ relative to EV from untreated MetS rats (Figure 2D,E).

### 3.3. RAS Component Proteins in EVs and Effects of MetS and Treatment with Candesartan

**Figure 3:**
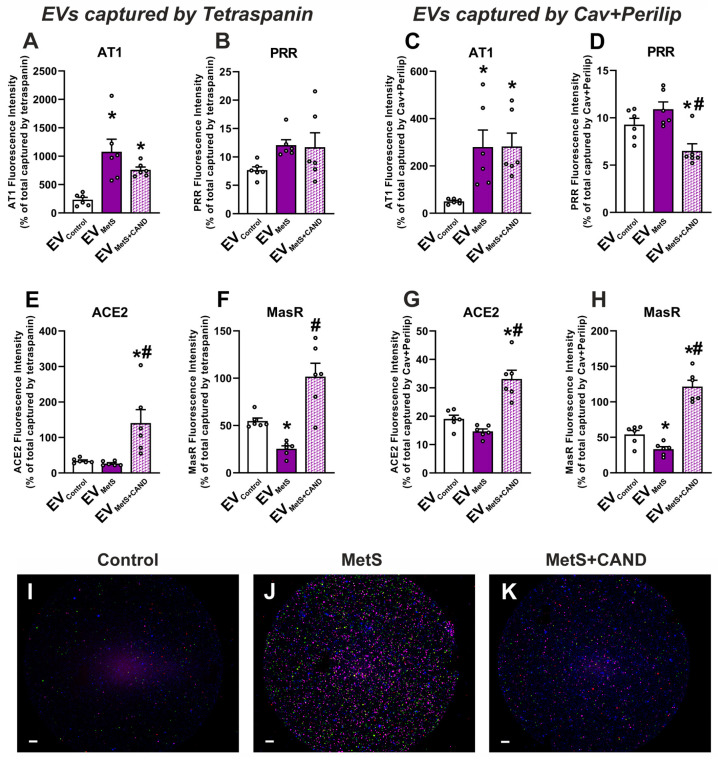
Analysis of EVs from rat serum using SP-IRIS/ExoView^®^. An increase in the fluorescence intensity of the pro-inflammatory AT1 receptor (**A**) and a non-significant trend towards increasing the fluorescence intensity of PRR (**B**) was observed in EV_MetS_ relative to EV_Control_ captured by tetraspanins (i.e., total serum EVs). Similarly, a significant increase in AT1 (**C**) and a non-significant increase in PRR (**D**) fluorescence was observed in EV_MetS_ when captured by Cav + Perlip (i.e., EVs from fat tissue). A tendency to decrease ACE2 levels (**E**,**G**), and a significant decrease in the levels of the anti-inflammatory Mas receptor was observed in EV_MetS_ relative to EV_Control_ both captured by tetraspanins (**E**,**F**) and by Cav + Perlip (**G**,**H**). Interestingly, EVs from MetS rats treated with candesartan (EV_MetS+CAND_) showed a significant increase in the fluorescence intensity of the anti-inflammatory components ACE2 (**E**,**G**) and MasR (**F**,**H**). Photographs (**I**–**K**) show label-free interferometry (IFM) images of a representative anti-Caveolin capture chip from control rats (**I**), MetS rats (**J**), and MetS rats treated with candesartan (**K**). Pink (AT1 receptor), green (PRR), blue (ACE2), and red (MasR) fluorescence can be observed. Scale bars: 10 µm. Data are given as the mean ± SEM. * *p* < 0.05 compared to the EV_Control_ group; # *p* < 0.05 compared to the EV_MetS_ group (Kruskal–Wallis one-way ANOVA with the Student-Newman-Keuls method as a post hoc test (**A**,**E**,**F**); one-way ANOVA with the Student-Newman-Keuls method as a post hoc test (**B**–**D**,**G**,**H**)). ACE2: angiotensin-converting enzyme 2; AT1: angiotensin type 1 receptor; CAND: candesartan; Cav: Caveolin; EVs: extracellular vesicles; EV_Control_: EVs isolated from the serum of control animals; EV_MetS_: EVs isolated from the serum of metabolic syndrome animals; EV_MetS+CAND_: EVs isolated from the serum of metabolic syndrome animals treated with candesartan; MasR: Mas-related receptor; MetS: metabolic syndrome; Perilip: Perilipin-1, PRR: (pro)-renin receptor.

First, we analyzed the presence of RAS proteins in serum EVs captured by tetraspanins (CD9, CD81; i.e., total serum EVs) measuring the fluorescence intensity of the secondary antibodies. Due to among-group differences in the number of captured particles, the values were normalized by the total number of particles captured from each animal sample. The results showed an increase in the fluorescence intensity of the pro-inflammatory AT1 receptors in EVs from MetS rats relative to EVs from control rats (Figure 3A) which reveals a higher AT1 receptor expression in EV_MetS_ relative to EV_Control_. The PRR receptor showed a non-significant trend towards increasing fluorescence intensity in EV_MetS_ (Figure 3B). A trend towards a decline in ACE2 (Figure 3E) and a significant decrease in MasR (Figure 3F) fluorescence was observed in EV_MetS_ relative to EV_Control_, which indicates a lower expression of the anti-inflammatory/anti-oxidative arm components in EV_MetS_. Interestingly, the treatment of MetS rats with candesartan induced a significant increase in the fluorescence intensity of components of the anti-oxidative/anti-inflammatory RAS such as ACE2 (Figure 3E) and MasR (Figure 3F) in EV_MetS+CAND_. Similar results were obtained in EVs captured by fat tissue markers (Caveolin and Perilipin-1; i.e., EVs from fat tissue), showing upregulation of the pro-oxidative/pro-inflammatory (Figure 3C,D) and downregulation of the anti-oxidative/anti-inflammatory (Figure 3G,H) RAS components in EV_MetS_. In EVs from rats treated with candesartan (EV_MetS+CAND_), a significant increase in the fluorescence for the anti-inflammatory ACE2 (Figure 3G) and MasR (Figure 3H) RAS components was observed. Label-free interferometry (IFM) images from the different experimental groups are shown in Figure 3I–K.

### 3.4. mRNA Expression of Pro-Inflammatory Cytokines and Pro-Oxidative Markers in Serum EVs and Effects of Treatment of MetS Rats with Candesartan

**Figure 4:**
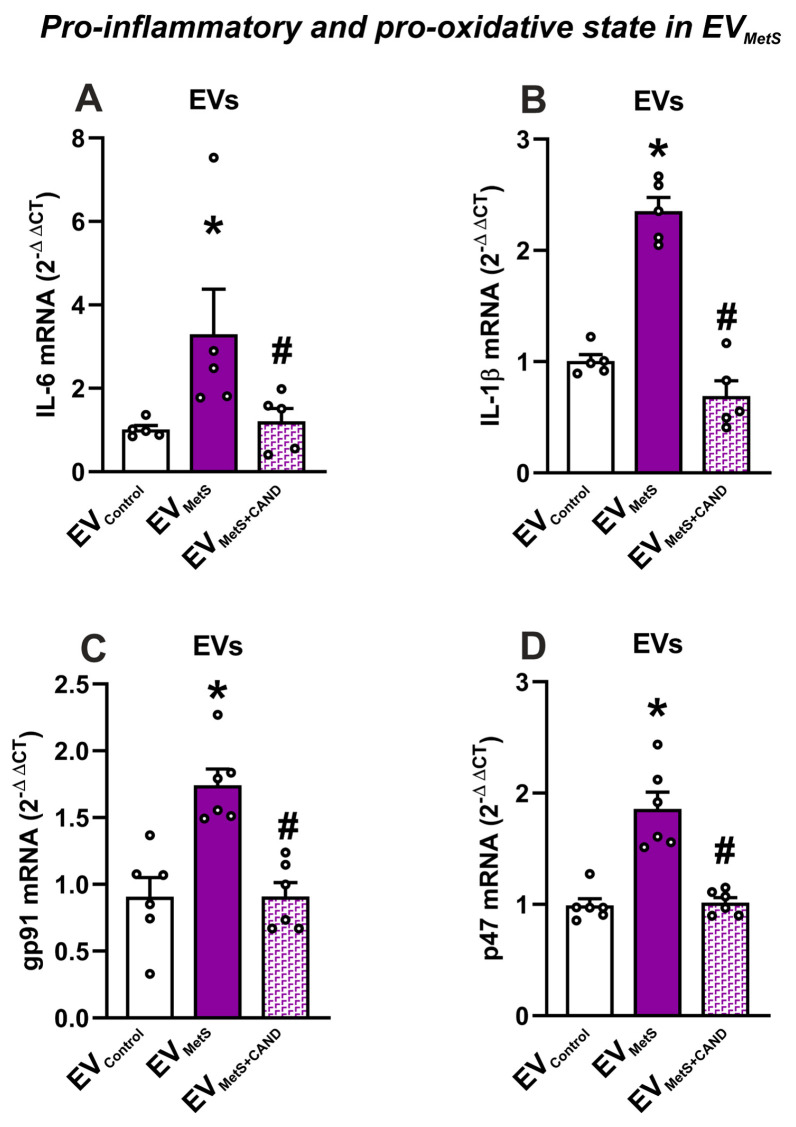
mRNA-cargo of pro-inflammatory and pro-oxidative markers in EVs. mRNA expressions of the pro-inflammatory interleukins IL-6 (**A**) and IL-1β (**B**), and the NADPH-oxidase gp91 (**C**) and p47 (**D**) subunits were increased in EV_MetS_ relative to EV_Control_. The MetS-induced changes were inhibited when the MetS rats were treated with candesartan. For mRNA, the comparative cycle threshold values method (2^−ΔΔCt^) was used. Data are given as the mean ± SEM. * *p* < 0.05 compared to the control; # *p* < 0.05 compared to the EV_MetS_ (Kruskal–Wallis one-way ANOVA with the Student-Newman-Keuls method as a post hoc test (**A**,**D**); one-way ANOVA with the Student-Newman-Keuls method as a post hoc test (**B**,**C**)). CAND: candesartan; EVs: extracellular vesicles; EV_Control_: EVs isolated from the serum of control animals; EV_MetS_: EVs isolated from the serum of metabolic syndrome animals; EV_MetS+CAND_: EVs isolated from the serum of metabolic syndrome animals treated with candesartan; IL: interleukin; MetS: metabolic syndrome.

The possible pro-inflammatory and pro-oxidative cargo of EV_MetS_ was confirmed by measuring the mRNA levels of the interleukins IL-6 and IL-1β and the NADPH-oxidase subunits p47phox and gp91phox, respectively. Both mRNA interleukin levels and NADPH-oxidase mRNA subunits were significantly increased in EV_MetS_ relative to EV_Control_ and EV_MetS+CAND_. (Figure 4).

Altogether, these results reveal the deregulation of RAS in EV_MetS_ towards an increase in the components of the pro-inflammatory RAS axis, probably as the result of deregulation in RAS components in the cell of origin. Consistent with this, we also observed the upregulation of pro-inflammatory interleukins and pro-oxidative markers. This dysregulation was improved when animals suffering MetS were treated with the AT1 receptor blocker candesartan, particularly by the upregulation of anti-inflammatory/anti-oxidative RAS components (i.e., the counterregulatory RAS axis).

### 3.5. Uptake of EV_MetS_ by the N27 Dopaminergic Cell Line and the C6 Astrocytic Cell Line In Vitro

**Figure 5:**
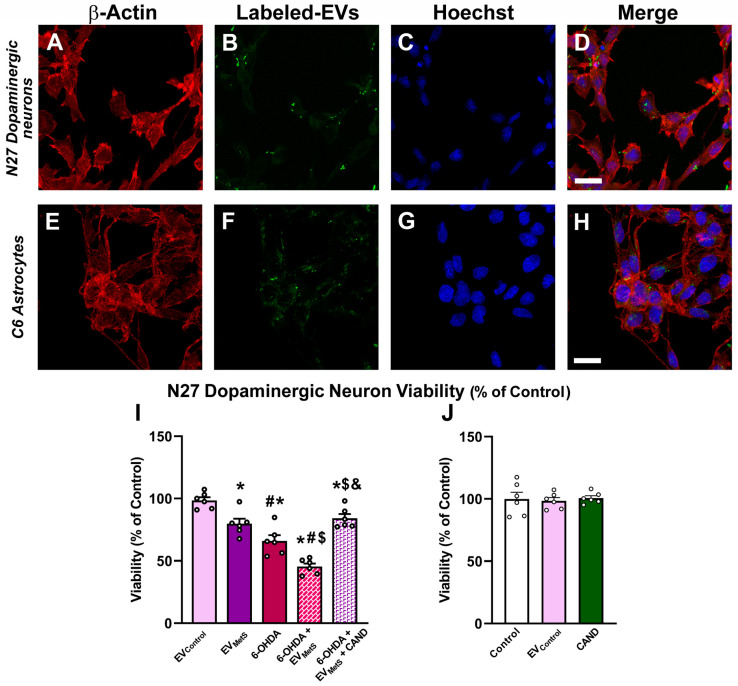
N27 dopaminergic cell line and C6 astrocytic cell line uptake of EV_MetS_. Triple fluorescent labeling for (red) the cell marker β-Actin (**A**,**E**), the labeled-EVs (green; (**B**,**F**)), and the nuclear marker (blue) Hoechst 33342 (**C**,**G**). Labeled EVs were uptaken by N27 (**D**) and C6 (**H**) cells. The MTT assay revealed that EV_MetS_ decreased the viability of the N27 dopaminergic cell line and that cultures simultaneously treated with 6-OHDA plus EV_MetS_ showed a significant decrease in cell viability relative to the cultures treated with 6-OHDA alone. The decrease in dopaminergic viability was significantly improved when cultures were previously treated with candesartan (**I**). However, dopaminergic viability was not affected by the treatment of the N27 dopaminergic cell line with the vehicle (control) or EVs from control rats (EV_Control_) or candesartan alone (**J**). Scale bar: 20 µm. Data are given as the mean ± SEM. * *p* < 0.05 compared to the EV_Control_ group; # *p* < 0.05 compared to the EV_MetS_ group; ^$^ *p* < 0.05 compared to the 6-OHDA group; ^&^ *p* < 0.05 compared to the 6-OHDA plus EV_MetS_ group, (one-way ANOVA with the Student-Newman-Keuls method as a post hoc test (**I**) and Kruskal–Wallis one-way ANOVA (**J**)). 6-OHDA: 6-hydroxydopamine; CAND: candesartan; EVs: extracellular vesicles; EV_Control_: EVs isolated from the serum of control rats; EV_MetS_: EVs isolated from the serum of rats with metabolic syndrome; MetS: metabolic syndrome.

To determine whether the N27 dopaminergic cell line and the C6 astrocytic cell line are targeted by EV_MetS_, an uptake study was designed using the N27 dopaminergic cell line and the C6 astrocytic cell line treated with ExoGlow™-labeled EVs. The cell cytoplasms were immunostained with an Anti-β-Actin antibody, the nuclei were counterstained with Hoechst 33342, and colocalization with EVs was investigated by confocal fluorescent microscopy. Our results showed that the labeled EVs were taken up by both the N27 dopaminergic cell line and C6 astrocytic cell line (Figure 5A–H).

### 3.6. EV_MetS_ Enhance N27 Dopaminergic Cell Line Death in Cell Cultures Mediated by AT1 Receptor Activation

As EVs were taken up by the N27 dopaminergic cell line, we studied whether EV_MetS_ internalization could induce an increase in dopaminergic neuron death. Cellular viability was measured using the MTT assay. Our results revealed that EV_MetS_ significantly reduced N27 dopaminergic cell line viability and that the cultures simultaneously treated with the 6-OHDA neurotoxin plus EV_MetS_ showed a significant decrease in cell survival relative to the cultures treated with 6-OHDA alone (Figure 5I). However, the increase in the dopaminergic vulnerability was significantly reduced when cultures were previously treated with the AT1 receptor blocker candesartan (Figure 5I), suggesting that EV_MetS_ can enhance the dopaminergic degeneration, which is mediated by AT1 receptor activation. No changes in N27 dopaminergic cell line viability were observed when cultures were treated with EV_Control_ or with Candesartan (Figure 5J).

### 3.7. EV_MetS_ Modulate the C6 Astrocytic Cell Line and N27 Dopaminergic Cell Line Function through AT1 Receptor Activation

**Figure 6:**
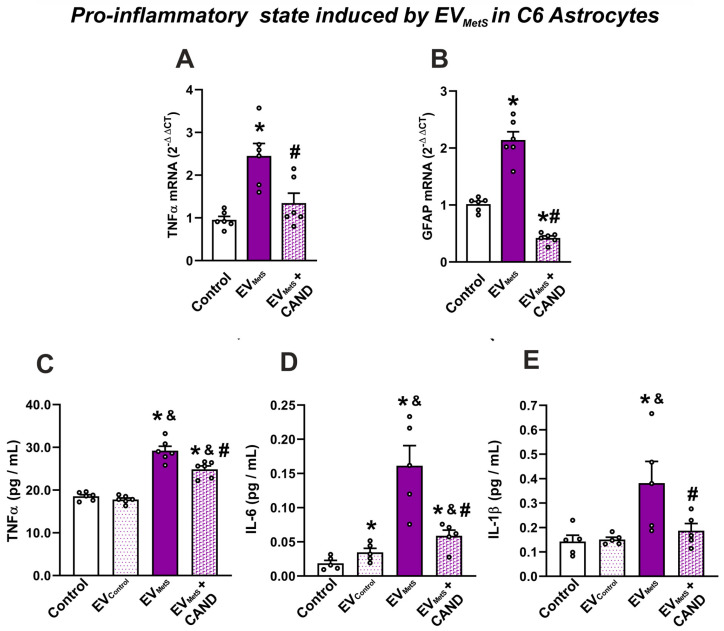
EV_MetS_ uptake increased the mRNA expression of TNFα (**A**) and GFAP (**B**) in C6 cells and the release of TNFα (**C**), IL-6 (**D**), and IL-1β (**E**) to the culture medium. The effects of EV_MetS_ were inhibited when cell cultures were simultaneously treated with the AT1 blocker candesartan. For mRNA, the comparative cycle threshold values method (2^−ΔΔCt^) was used. Data are given as the mean ± SEM. * *p* < 0.05 compared to the control group (i.e., cultures treated with the vehicle); # *p* < 0.05 relative to the EV_MetS_ group; ^&^ *p* < 0.05 compared to EV_Control_ (one-way ANOVA with the Student-Newman-Keuls Method as a post hoc test (**A**–**C**); Kruskal–Wallis one-way ANOVA with the Student-Newman-Keuls Method as a post hoc test (**D**,**E**)). CAND: candesartan, EVs: extracellular vesicles; EV_Control_: EVs isolated from the serum of control rats; EV_MetS_: EVs isolated from the serum of metabolic syndrome animals; GFAP: glial fibrillary acidic protein; IL: interleukin, MetS: metabolic syndrome; TNFα: Tumor necrosis factor-alpha.

The uptake of EV_MetS_ by the C6 astrocytic cell line upregulated the mRNA expression of GFAP and TNF-α in cells, suggesting that EV_MetS_ promote astrocyte pro-inflammatory responses. However, the administration of candesartan to cultures significantly inhibited the increase in GFAP and in TNF-α mRNA levels induced by EV_MetS_, suggesting that EV_MetS_ modify the C6 astrocytic cell line function and that this is mediated by AT1 receptor activation. (Figure 6A,B). Furthermore, EV_MetS_ induced an intense IL-1β, IL-6, and TNF-α release to the culture medium, which was inhibited in the presence of candesartan (Figure 6C–E).

**Figure 7:**
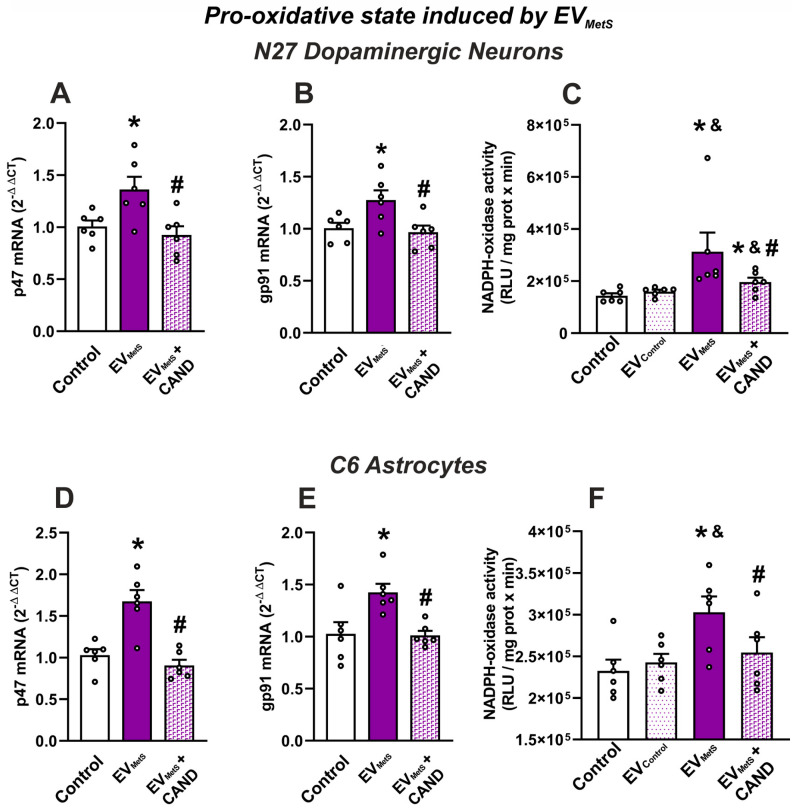
Oxidative stress induced by EV_MetS_ in the N27 dopaminergic cell line and the C6 astrocytic cell line. The increased mRNA expression of p47phox (**A**,**D**) and gp91phox NADPH-oxidase subunits (**B**,**E**) and NADPH-oxidase activity (**C**,**F**) induced by EV_MetS_ uptaking was inhibited when cells were treated with candesartan. For mRNA, the comparative cycle threshold values method (2^−ΔΔCt^) was used. NADPH oxidase activity was expressed as relative light units (RLU)/mg protein × min). Data are given as the mean ± SEM. * *p* < 0.05 relative to the control group; # *p* < 0.05 compared to the EV_MetS_ group; ^&^ *p* < 0.05 compared to EV_Control_ (One-way ANOVA with the Student-Newman-Keuls Method as a post hoc test (**A**,**B**,**D**–**F**); Kruskal–Wallis one-way ANOVA with the Student-Newman-Keuls Method as a post hoc test (**C**)). CAND: candesartan; control group: cultures treated with vehicle; EV_Control_: EVs isolated from the serum of control rats; EV_MetS_: EVs isolated from the serum of metabolic syndrome animals; MetS: metabolic syndrome.

To study the possible role of EV_MetS_ as oxidative stress inducers, NADPH-oxidase activity, and mRNA levels of NADPH-oxidase subunits gp91phox and p47phox were measured in the N27 dopaminergic cell line and the C6 astrocytic cell line, as a major cell source of superoxide and oxidative stress. Our results showed that treatment with EV_MetS_ enhanced the NADPH-oxidase activity and mRNA levels of gp91phox and p47phox in both cell types relative to control cultures (Figure 7). However, cells treated with candesartan plus EV_MetS_ showed a significant decrease in NADPH-oxidase activity and mRNA levels of gp91phox and p47phox relative to those treated with EV_MetS_ alone suggesting that the increase in NADPH-oxidase activity is mediated by activation of AT1 receptor, (Figure 7).

### 3.8. EV_MetS_ Uptake Leads to RAS Dysregulation in Cultured N27 Dopaminergic Cell Line and C6 Astrocytic Cell Line

**Figure 8:**
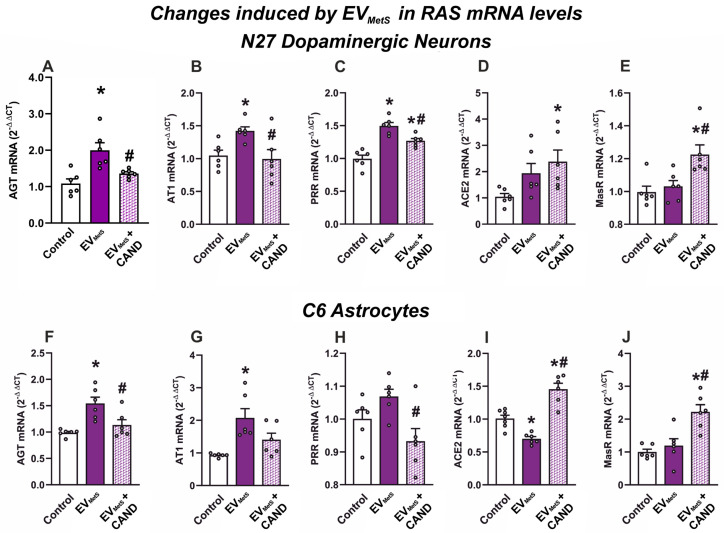
RAS dysregulation induced by EV_MetS_ in the N27 dopaminergic cell line and the C6 astrocytic cell line. The increase in the mRNA expression of the pro-inflammatory RAS components AGT (**A**,**F**), AT1 (**B**,**G**), and PRR (**C**,**H**) induced by EV_MetS_ uptaking was inhibited when cells were treated with candesartan. The mRNA expression of the anti-inflammatory RAS components ACE2 (**D**,**I**) and MasR (**E**,**J**) was increased after candesartan treatment. For mRNA, the comparative cycle threshold values method (2−^ΔΔCt^) was used. Data are given as the mean ± SEM. * *p* < 0.05 compared to the control group; # *p* < 0.05 compared to the EV_MetS_ group (one-way ANOVA with the Student-Newman-Keuls Method as a post hoc test (**A**–**D**,**F**,**H**–**J**); Kruskal–Wallis one-way ANOVA with the Student-Newman-Keuls method (**E**) and Dunn’s method (**G**) as post hoc tests). ACE2: angiotensin-converting enzyme 2; AGT: angiotensinogen; AT1: angiotensin type 1 receptor; CAND: candesartan; EVs: extracellular vesicles; EV_MetS_: EVs isolated from the serum of metabolic syndrome animals; MasR: Mas-related receptor; MetS: metabolic syndrome; PRR: (pro)-renin receptor.

To know if the abovementioned effects of EV_MetS_ on the N27 dopaminergic cell line and the C6 astrocytic cell line may be mediated by cell RAS dysregulation, the mRNA levels of different RAS components were measured in both the N27 dopaminergic cell line and the C6 astrocytic cell line. Our results showed a significant increase in the mRNA expression of components of the pro-inflammatory RAS axis (AGT, AT1, and PRR) that was not found when cells were treated with candesartan (Figure 8A–C,F–H). Furthermore, N27 and C6 cells showed a significant increase in the mRNA levels of the RAS anti-inflammatory components after candesartan treatment (Figure 8D,E,I,J). In summary, these results strongly suggest that the uptake of EV_MetS_ induces the dysregulation of cell RAS towards the pro-oxidative/pro-inflammatory axis in recipient cells, which is inhibited when cultures are pre-treated with the AT1 receptor blocker candesartan.

## 4. Discussion

The present results show that the number of EVs is highly increased in the serum of MetS rats, and this increase is inhibited in MetS rats treated with the AT1 receptor blocker candesartan. Furthermore, we found that EVs from MetS rats (EV_MetS_) have elevated levels of pro-oxidative/pro-inflammatory RAS axis components, indicating that the MetS may alter the concentration and composition of circulating EVs. The results in rat models also show that treatment with EV_MetS_ decreases the viability of the N27 dopaminergic cell line and modulates C6 astrocytic cell line function, upregulating markers of neuroinflammation and oxidative stress, which is inhibited by treatment with candesartan.

MetS patients showing three of the pathological conditions that characterize the disease have a 31% higher risk of PD than individuals with none of the factors, and patients showing five of the MetS criteria have a 66% higher risk of PD [6]. It was also observed that MetS increases the risk of cognitive impairment in PD patients [8]. However, the mechanisms linking MetS and PD have not been clarified. It is currently accepted that obesity is an important factor in the development of MetS. In obesity, adipocytes suffer hypertrophic and hyperplasic growth and are under stress [30,53]. The RAS is present in adipose tissue [54] and the production of RAS components by adipocytes is dysregulated by obesity, contributing to dysregulation of the systemic RAS and its consequences such as hypertension, insulin resistance in diabetes, inflammation, and oxidative stress [55]. Consistent with this, the treatment of MetS patients with the AT1 blocker irbesartan induced a significant reduction of blood pressure together with a reduction in cardiovascular risk factors: HDL cholesterol, serum triglycerides, fasting blood glucose, and waist circumference [56]. MetS-induced RAS dysregulation may also contribute to the progression of PD. Several studies in animal models of Parkinson´s disease show that overactivation of the pro-oxidative RAS axis in the substantia nigra and striatum (i.e., upregulation of the Ang II/AT1 axis) plays a major role in the progression of dopaminergic neuron degeneration via enhancement of oxidative stress and neuroinflammation [16]. Interestingly, the major role of AT1 overactivity in dopaminergic degeneration has been further supported by a recent study using single-cell genomic profiling of human dopaminergic neurons that identified the high expression of AT1 receptor gene (*Agtr1*) as the best marker for the most vulnerable neurons in humans, including PD patients [14]. Consistent with this, two recent clinical studies have shown the neuroprotective effects of treatment with AT1 blockers in PD [57,58].

Recent studies have revealed that a MetS-induced increase in agonistic AT1 autoantibodies may be a linking mechanism between MetS and PD [7]. However, the present results show that EVs released from adipocytes, and particularly the RAS cargo in those EVs, may also constitute a major linking mechanism between MetS and PD, as previously shown between MetS and several peripheral diseases. In obesity, the production of EVs is increased, which has been correlated with the onset of obesity-related diseases such as insulin resistance [59], metabolic dysfunction [30], and cancer [60]. Our results are consistent with these previous results and show an important increase in the number of EVs captured both by tetraspanins (i.e., total EVs) and by caveolin/perilipin (i.e., EVs enriched from fat tissue origin), which reveals an increase in EVs released from the fat tissue. Interestingly, this increase is inhibited in animals treated with the AT1 receptor blocker, candesartan. The mechanisms linking RAS and the increase in EV release remain to be fully clarified. However, previous studies have shown an increase in phosphorylated myosine phosphatase targeting protein-1 (p-MYPT1) and in Rho-associated kinase activity in fat tissue from animals subjected to a high-fat diet [30,61], which has been shown to be necessary and sufficient for the formation of membrane blebs [62,63] that is an essential step before EV formation. Interestingly, the activation of the Ang II/AT1/NADPH-oxidase axis induces Rho-kinase (ROCK) activation [64,65], and AT1 receptor blocking has been shown to be effective in inhibiting Rho kinase activity [64,66]. Consistent with this, the overactivity of the Ang II/AT1 pro-inflammatory axis may induce an increase in the number of released EVs in MetS animals, which is inhibited by the AT1 receptor blocker candesartan, as observed in the present study. However, additional mechanisms may also be involved. We have also observed a significant increase in the size of EVs from MetS rats, which was not inhibited by treatment with candesartan. This agrees with the idea that in fat tissue, in addition to the pro-inflammatory state of the origin cell [67], the size of EVs depends on other factors such as the nutritional status [43].

EV content reflects the physiological state of the origin cell [68], which includes the levels of RAS components in EVs from adipocytes [69]. In adipose tissue from animals treated with obesogenic diets, previous studies have shown an increase in AT1 receptor expression [70], an increase in PRR expression [55], and an increase in levels of AGT [71,72]. This is consistent with the present results showing the dysregulation of RAS components towards an increase in the pro-inflammatory component axis in EV_MetS_. We observed an increase in AT1, PRR, and AGT mRNA expression in EV_MetS_, which was decreased by treatment of MetS rats with candesartan. On the other hand, a significant decrease was observed in the expression of EV_MetS_ mRNA for MasR, a major component of the anti-inflammatory RAS axis, which was inhibited in MetS rats treated with candesartan. The dysregulation of adipose tissue MasR in obesity is consistent with recent studies showing that mice lacking MasR are prone to obesity and altered metabolism [73]. We did not detect significant changes in ACE2 mRNA levels in MetS rats. Changes in protein levels, as detected with the SP-IRIS/ExoView technology, in different RAS components revealed changes similar to those observed for the mRNA cargo (i.e., MetS-induced upregulation of pro-oxidative pro-inflammatory and downregulation of anti-oxidative anti-inflammatory RAS cargo, which was reverted by candesartan treatment), with minor differences possibly related to methodological reasons, such as a candesartan-induced increase in ACE2 protein levels not observed for ACE2 mRNA expression.

In addition to the observation of a dysregulation in the EV_MetS_ cargo towards the upregulation of the RAS pro-inflammatory axis, we confirmed the pro-inflammatory and pro-oxidative state of EV_MetS_ by measuring the mRNA expression for the interleukins IL-6 and IL-1β and the NADPH-oxidase subunits p47phox and gp91phox, which were significantly increased in EV_MetS_ rats relative to EVs from control rats and candesartan-treated MetS rats. This is consistent with previous data from adipose tissue of genetically obese diabetic KKAy mice, showing that the AT1 receptor blocker olmesartan induced a suppressive effect on IL-6 mRNA expression and decreased the oxidative stress (measured by mRNA expression of NADPH-oxidase subunits gp91phox and p47phox as in the present experiments) [74].

After the characterization of the EV_MetS_, showing upregulation of the pro-inflammatory RAS components and their pro-oxidative pro-inflammatory condition, we assumed that they could cross the BBB and access the brain, as shown by several previous studies [35,36]. Thus, we studied the effects of EV_MetS_ on N27 dopaminergic cell line degeneration and C6 astrocytic cell line function. Astrocytes are particularly relevant for the present study, as they both play a major role in dopaminergic degeneration [23] and are the major producers of AGT for the brain paracrine RAS [20,21,22]. Using the N27 dopaminergic cell line and the C6 astrocytic cell line, we confirmed that EV_MetS_ are uptaken by the N27 dopaminergic cell line and the C6 astrocytic cell line. Furthermore, we showed that this uptake reduces N27 dopaminergic cell line viability in cultures and enhances the dopaminergic neuron death triggered by the neurotoxin 6-OHDA, which is inhibited by simultaneous treatment with candesartan. Our results in cultures confirm that EV_MetS_ can directly exert deleterious effects in the N27 dopaminergic cell line and that AT1 receptor overactivation/overexpression mediates this effect, as the increase in N27 dopaminergic cell line death and the increase in the mRNA expression of components of the pro-inflammatory RAS axis (AGT, AT1, and PRR) were not observed when cells were treated with candesartan. Furthermore, N27 cells treated with EV_MetS_ plus candesartan showed an increase in the mRNA levels of components of the RAS anti-inflammatory axis. A similar regulation of RAS components was observed in cardiomyocytes after treatment with EVs from cardiac fibroblasts [34]. The exact mechanism linking EV_MetS_ and the upregulation of the N27 dopaminergic cell line pro-inflammatory RAS requires future studies specifically focused on this point. However, it was shown that after the release of mRNA cargo inside the recipient cell, EVs can regulate gene expression through de novo translation and post-translational regulation of target mRNA [75]. It has been suggested that the changes may be mediated by regulating the intracellular or intracrine RAS [32], and dopaminergic neurons have an intraneuronal RAS that we have described in detail in previous studies [17].

The deleterious effects EV_MetS_, after neuronal RAS dysregulation towards the pro-oxidative/pro-inflammatory state, are consistent with previous studies showing the enhancing effect of AT1 overactivity on dopaminergic degeneration [16] and the neuroprotective effects of AT1 receptor blockers (ARBs) in cellular, animal [16] models of Parkinson´s disease, and clinical studies [57,58]. It is known that there are RAS receptors in different types of neurons, and that brain RAS is involved in different brain functions [19,76]. In addition, other recent studies have shown that adipose-tissue-derived EVs can be transferred to different neuron types such as hippocampal neurons [77]. Therefore, MetS EVs induced RAS dysregulation may affect other neuronal populations and other brain functions. However, the present study has been focused on dopaminergic neurons, as MetS has been associated with a higher risk of PD [6], and there is an important effect of RAS dysregulation on dopaminergic neuron vulnerability [15,17] and AT1 gene overexpression identifies the most vulnerable dopaminergic neurons located in the ventral substantia nigra in humans, while less vulnerable dopaminergic neurons in the dorsal substantia nigra showed very low levels of AT1 gene expression [14].

The uptake of EV_MetS_ by C6 cells induced a strong increase in GFAP and TNF-α mRNA expression, suggesting that EV_MetS_ promote astroglial pro-inflammatory responses, an event that has been extensively associated with the progression of Parkinson’s disease [23]. The present results suggest that EV_MetS_ also upregulate other markers of the astrocytic inflammatory response, such as changes in the astrocytic morphology, which may be analyzed in future studies. The effects of EV_MetS_ uptake were significantly inhibited by candesartan, which is consistent with previous results showing that AT1 receptor blockade prevents astrocyte activation [78]. Furthermore, it has been shown that aging and inflammatory stimuli [79] induce astrocytic secretion of EVs [80,81] loaded with proteins and nucleic acids involved in inflammatory signal transmission, neurotrophic signals, and modulation of neuronal excitability [82,83,84]. In line with these results, we observed an intense release of IL-1β, IL-6, and TNF-α to the culture medium that was inhibited in the presence of candesartan. As in the case of the N27 dopaminergic cell line, the intracellular events linking EV_MetS_ and astrocyte pro-inflammatory RAS activation remain to be elucidated in future specific studies.

Consistent with the abovementioned in vitro results showing EV_MetS_-induced Ang II/AT1 overactivity, treatment with EV_MetS_ also enhanced the oxidative stress by increasing the NADPH-oxidase activity and mRNA levels of gp91phox and p47phox in the N27 dopaminergic cell line and in the C6 astrocytic cell line relative control cells, as it is known that NADPH-oxidase activation is a major downstream mechanism for the AT1 effects [65]. Consistent with this, cells treated with candesartan plus EV_MetS_ showed a significant decrease in NADPH-oxidase activity and the mRNA levels of gp91phox and p47phox relative to those treated with EV_MetS_ alone. Similar upregulation of oxidative stress markers was observed in mouse aorta after administration of EVs from MetS patients [85].

## 5. Conclusions

In conclusion, our results in rat models show that MetS may generate circulating EV_MetS_ that may increase the progression of neuroinflammation and dopaminergic neurodegeneration through RAS dysregulation in recipient cells, and that this process can be inhibited by treatment with AT1 receptor blockers.

## Figures and Tables

**Table 1:** Primer sequences for RT-PCR.

Genes	Primers (5′-3′)	Accession Number	Product Length (bps)
*Ace2*	F: GTGGAGGTGGATGGTCTTTCAGG; R: CACCAACGATCTCCCGCTTCA	NM_001012006.2	86
*Actb*	F: TCGTGCGTGACATTAAAGAG; R: TGCCACAGGATTCCATACC	NM_031144.3	198
*Agt*	F: GAGTGAGGCAAGAGGTGTA; R: TCCAACGATCCAAGGTAGAA	NM_134432.2	90
*Agtr1a*	F: GCCAAGCCAGCCATCAGC; R: TTCAACCTCTACGCCAGTGTG;	NM_030985.4	149
*Atp6ap2* (*Prr*)	F: TGGTGGGAATGCAGTGGTAGAG; R: GGGACTTTGGGTGTTCTCTTGTT	NM_001007091.1	109
*Cybb* (*Gp91-phox*)	F: ATCTTGCTGCCAGTGTGTCG; R: AATGGTGTGAATGGCCGTGTG	NM_023965.2	153
*Gapdh*	F: ACATACTCAGCACCAGCAT; R: GCAAGTTCAACGGCACAGT	NM_017008.4	*124*
*Gfap*	F: TGGAGGTGGAGAGGGACAATC; R: CTCCAGATCCACACGAGCCAA	NM_017009.2	152
*Il1b*	F: GGCAACTGTCCCTGAACTCA; R: TGTCGAGATGCTGCTGTGAGA	NM_031512.2	170
*Il6*	F: TGGTATCCTCTGTGAAGTCTC; R: CAGCCAGTTGCCTTCTTG	NM_012589.2	91
*Mas1*	F: TCCCCAGACCAGTCATCCT; R: TGCTGGAGGTATTCATGGCTT	NM_012757.2	122
*Ncf1* (*P47-phox*)	F: TGTTCCTGGTTAAGTGGCAGGA; R: CTGGGAGCTGGGAGGTGAG	NM_053734.2	157
*Tnf*	F: CACGTCGTAGCAAACCACCA; R: GGTTGTCTTTGAGATCCATGCCA	NM_012675.3	97

## Data Availability

The datasets used and/or analyzed during the current study are available from the corresponding author on reasonable request.

## Supplementary Materials

The following supporting information can be downloaded at: https://www.mdpi.com/article/10.3390/antiox12122045/s1. Figure S1: Additional control experiments. Photomicrographs of representative dopaminergic cell line N27 (**A**; TH-positive, green) and C6 astrocytic cell line (**B**; GFAP-positive, green) show that the cell lines express the phenotype of dopaminergic neurons and astrocytes. Nuclei are labeled in blue with Hoechst. Labelling was carried out as previously described [20]. Scale bar: 5 µm. Effects of treatment with EV_Control_ in N27 dopaminergic cell line (**C**) and C6 astrocytic cell line (**D**). No changes were observed after treatment with EV_Control_ relative to Control (i.e. cultures treated with EV vehicle, PBS). Data are given as mean ± SEM. All comparisons were carried out using the Student t-test, except for AT1 in the N27 cell line, where the Mann-Whitney Rank Sum Test was used (*n* = 3–4/group). ACE2: angiotensin-converting enzyme 2; AGT: angiotensinogen; AT1: angiotensin type 1 receptor; EV_Control_: EVs isolated from the serum of control animals; MasR: Mas-related receptor; PRR: (pro )-renin receptor. Table S1: Results of Statistical Analysis.
