# Supplementary material for: Extracellular Vesicles and Their Renin–Angiotensin Cargo as a Link between Metabolic Syndrome and Parkinson’s Disease
Source: Antioxidants (Basel). 2023 Nov 26;12(12):2045. doi: 10.3390/antiox12122045 (PMC10741149; doi:10.3390/antiox12122045)
Supplement: Supplementary file 1 [file antioxidants-12-02045-s001.zip › antioxidants-2705783-supplementary.pdf]

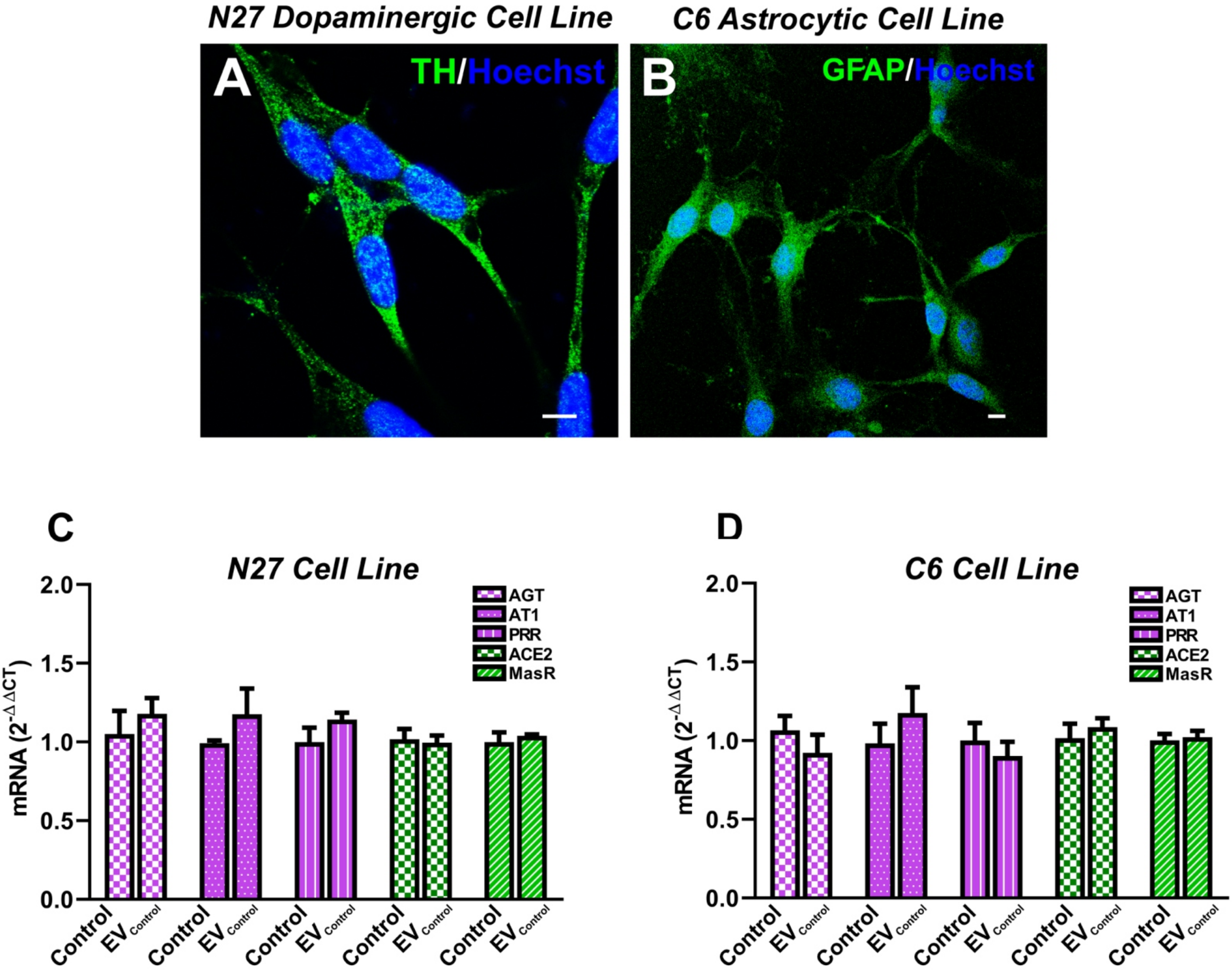


**Figure S1.** Additional control experiments. Photomicrographs of representative dopaminergic cell line N27 (**A**; TH-positive, green) and C6 astrocytic cell line (**B**; GFAP-positive, green) show that the cell lines express the phenotype of dopaminergic neurons and astrocytes. Nuclei are labeled in blue with Hoechst. Labelling was carried out as previously described [20]. Scale bar: 5 µm. Effects of treatment with $EV_{Control}$ in N27 dopaminergic cell line (**C**) and C6 astrocytic cell line (**D**). No changes were observed after treatment with $EV_{Control}$ relative to Control (i.e. cultures treated with EV vehicle, PBS). Data are given as mean ± SEM. All comparisons were carried out using the Student t-test, except for AT1 in the N27 cell line, where the Mann-Whitney Rank Sum Test was used (n=3-4/group). ACE2: angiotensin-converting enzyme 2; AGT: angiotensinogen; AT1: angiotensin type 1 receptor; $EV_{Control}$: EVs isolated from the serum of control animals; MasR: Mas-related receptor; PRR: (pro)-renin receptor.

**Table S1.** Results of Statistical Analysis

| Figure | Groups | Statistical test | Statistics | p value |
|---|---|---|---|---|
| **1C** | Control *vs* MetS *vs* MetS+CAND | Kruskal-Wallis One Way Analysis of Variance on Ranks | H(2)= 11.942 | 0.003 |
| **1D** | Control *vs* MetS *vs* MetS+CAND | One Way Analysis of Variance | $F_{(2,15)}$= 18.955 | <0.001 |
| **2A** | Control *vs* MetS *vs* MetS+CAND | Kruskal-Wallis One Way Analysis of Variance on Ranks | H(2)= 12,784 | 0,002 |
| **2B** | Control *vs* MetS *vs* MetS+CAND | One Way Analysis of Variance | $F(_{2,14})$= 28,116 | <0,001 |
| **2C** | Control *vs* MetS *vs* MetS+CAND | One Way Analysis of Variance | $F(_{2,14})$= 11,980 | <0.001 |
| **2D** | Control *vs* MetS *vs* MetS+CAND | One Way Analysis of Variance | $F(_{2,15})$= 0,924 | 0.418 |
| **2E** | Control *vs* MetS *vs* MetS+CAND | One Way Analysis of Variance | $F(_{2,13})$= 4,721 | 0,029 |
| **3A** | Control *vs* MetS *vs* MetS+CAND | Kruskal-Wallis One Way Analysis of Variance on Ranks | H(2)= 11.789 | 0.003 |
| **3B** | Control *vs* MetS *vs* MetS+CAND | One Way Analysis of Variance | $F(_{2,15})$= 2.369 | 0.128 |
| **3C** | Control *vs* MetS *vs* MetS+CAND | One Way Analysis of Variance | $F(_{2,15})$= 24,197 | <0,001 |
| **3D** | Control *vs* MetS *vs* MetS+CAND | One Way Analysis of Variance | $F(_{2,15})$= 9,608 | 0.002 |
| **3E** | Control *vs* MetS *vs* MetS+CAND | Kruskal-Wallis One Way Analysis of Variance on Ranks | H(2)= 12.538 | 0.002 |
| **3F** | Control *vs* MetS *vs* MetS+CAND | Kruskal-Wallis One Way Analysis of Variance on Ranks | H(2)= 13,053 | 0.001 |
| **3G** | Control *vs* MetS *vs* MetS+CAND | One Way Analysis of Variance | $F(_{2,15})$= 24,197 | <0,001 |
| **3H** | Control *vs* MetS *vs* MetS+CAND | One Way Analysis of Variance | $F(_{2,15})$= 52,772 | <0,001 |
| **4A** | Control *vs* MetS *vs* MetS+CAND | Kruskal-Wallis One Way Analysis of Variance on Ranks | H(2)= 8,180 | 0.017 |
| **4B** | Control *vs* MetS *vs* MetS+CAND | One Way Analysis of Variance | $F(_{2,12})$= 62,291 | <0.001 |
| **4C** | Control *vs* MetS *vs* MetS+CAND | One Way Analysis of Variance | $F(_{2,15})$= 15.048 | <0.001 |
| **4D** | Control *vs* MetS *vs* MetS+CAND | Kruskal-Wallis One Way Analysis of Variance on Ranks | H(2)= 11.380 | 0.003 |
| **5I** | $EV_{Control}$ *vs* $EV_{MetS}$ *vs* 6-0HDA *vs* 60HDA+ $EV_{MetS}$ *vs* 60HDA+ $EV_{MetS}$+CAND | One Way Analysis of Variance | $F(_{4,25})$= 32.781 | <0.001 |
| **5J** | Control *vs* $EV_{Control}$ *vs* CAND | Kruskal-Wallis One Way Analysis of Variance on Ranks | H(2)= 0.363 | 0.834 |
| **6A** | Control *vs* $EV_{MetS}$ *vs* $EV_{MetS}$+CAND | One Way Analysis of Variance | $F(_{2,15})$= 12.651 | <0.001 |
| **6B** | Control *vs* $EV_{MetS}$ *vs* $EV_{MetS}$+CAND | One Way Analysis of Variance | $F(_{2,15})$= 90.922 | <0.001 |
| **6C** | Control *vs* $EV_{Control}$ *vs* $EV_{MetS}$ *vs* $EV_{MetS}$+CAND | One Way Analysis of Variance | F(3,20)= 59.941 | <0.001 |
| **6D** | Control *vs* $EV_{Control}$ *vs* $EV_{MetS}$ *vs* $EV_{MetS}$+CAND | Kruskal-Wallis One Way Analysis of Variance on Ranks | H(3)= 15.754 | 0.001 |
| **6E** | Control *vs* $EV_{Control}$ *vs* $EV_{MetS}$ *vs* $EV_{MetS}$+CAND | Kruskal-Wallis One Way Analysis of Variance on Ranks | H(3)= 9.025 | 0.029 |
| **7A** | Control *vs* $EV_{MetS}$ *vs* $EV_{MetS}$+CAND | One Way Analysis of Variance | $F(_{2,15})$= 6.470 | 0.009 |
| **7B** | Control *vs* $EV_{MetS}$ *vs* $EV_{MetS}$+CAND | One Way Analysis of Variance | $F(_{2,15})$= 5.584 | 0.015 |
| **7C** | Control *vs* $EV_{Control}$ *vs* $EV_{MetS}$ *vs* $EV_{MetS}$+CAND | Kruskal-Wallis One Way Analysis of Variance on Ranks | H(3)= 14.820 | 0.002 |
| **7D** | Control *vs* $EV_{MetS}$ *vs* $EV_{MetS}$+CAND | One Way Analysis of Variance | $F(_{2,15})$= 18.527 | <0.001 |

**Table S1.** Results of Statistical Analysis

| | | | | |
|---|---|---|---|---|
| **7E** | Control *vs* $EV_{MetS}$ *vs* $EV_{MetS}$+CAND | One Way Analysis of Variance | $F_{(2,15)}$= 8.013 | 0.004 |
| **7F** | Control *vs* $EV_{Control}$ *vs* $EV_{MetS}$ *vs* $EV_{MetS}$+CAND | One Way Analysis of Variance | $F_{(3,20)}$= 3.999 | 0.022 |
| **8A** | Control *vs* $EV_{MetS}$ *vs* $EV_{MetS}$+CAND | One Way Analysis of Variance | $F_{(2,15)}$= 10.692 | 0.001 |
| **8B** | Control *vs* $EV_{MetS}$ *vs* $EV_{MetS}$+CAND | One Way Analysis of Variance | $F_{(2,15)}$= 5.323 | 0.018 |
| **8C** | Control *vs* $EV_{MetS}$ *vs* $EV_{MetS}$+CAND | One Way Analysis of Variance | $F_{(2,15)}$= 27.324 | 0.001 |
| **8D** | Control *vs* $EV_{MetS}$ *vs* $EV_{MetS}$+CAND | One Way Analysis of Variance | $F_{(2,15)}$= 3.982 | 0.041 |
| **8E** | Control *vs* $EV_{MetS}$ *vs* $EV_{MetS}$+CAND | Kruskal-Wallis One Way Analysis of Variance on Ranks | H(2)= 8.082 | 0.018 |
| **8F** | Control *vs* $EV_{MetS}$ *vs* $EV_{MetS}$+CAND | One Way Analysis of Variance | $F_{(2,15)}$= 9.911 | 0.002 |
| **8G** | Control *vs* $EV_{MetS}$ *vs* $EV_{MetS}$+CAND | Kruskal-Wallis One Way Analysis of Variance on Ranks | H(2)= 9.739 | 0.008 |
| **8H** | Control *vs* $EV_{MetS}$ *vs* $EV_{MetS}$+CAND | One Way Analysis of Variance | $F_{(2,15)}$= 5.180 | 0.019 |
| **8I** | Control *vs* $EV_{MetS}$ *vs* $EV_{MetS}$+CAND | One Way Analysis of Variance | $F_{(2,16)}$= 36.022 | <0.001 |
| **8J** | Control *vs* $EV_{MetS}$ *vs* $EV_{MetS}$+CAND | One Way Analysis of Variance | $F_{(2,15)}$= 13.221 | <0.001 |
